# Emerging Treatment Options for Infections by Multidrug-Resistant Gram-Positive Microorganisms

**DOI:** 10.3390/microorganisms8020191

**Published:** 2020-01-30

**Authors:** Despoina Koulenti, Elena Xu, Andrew Song, Isaac Yin Sum Mok, Drosos E. Karageorgopoulos, Apostolos Armaganidis, Sotirios Tsiodras, Jeffrey Lipman

**Affiliations:** 1 UQ Centre for Clinical Research, Faculty of Medicine, The University of Queensland, Brisbane, QLD 4072, Australia; elena.xu@uq.net.au (E.X.); a.song@uq.net.au (A.S.); isaac.mok1@uq.net.au (I.Y.S.M.); j.lipman@uq.edu.au (J.L.); 2 2nd Critical Care Department, Attikon University Hospital, 12462 Athens, Greece; aarmag@med.uoa.gr; 3 4th Department of Internal Medicine, Attikon University Hospital, 12462 Athens, Greece; drkarag@gmail.com (D.E.K.); sotirios.tsiodras@gmail.com (S.T.); 4 Department of Intensive Care Medicine, Royal Brisbane and Women’s Hospital, Brisbane, QLD 4029, Australia; 5 Anesthesiology and Critical Care, Centre Hospitalier Universitaire De Nîmes (CHU), University of Montpellier, 30029 Nîmes, France

**Keywords:** emerging anti gram-positive antibiotics, multi-drug resistance organisms, clinical trials, dihydrofolate reductase inhibitors, ketolides, oxazolidinones, quinolones, defensin mimetics, β-lactams, topoisomerase II inhibitors

## Abstract

Antimicrobial agents are currently the mainstay of treatment for bacterial infections worldwide. However, due to the increased use of antimicrobials in both human and animal medicine, pathogens have now evolved to possess high levels of multi-drug resistance, leading to the persistence and spread of difficult-to-treat infections. Several current antibacterial agents active against Gram-positive bacteria will be rendered useless in the face of increasing resistance rates. There are several emerging antibiotics under development, some of which have been shown to be more effective with an improved safety profile than current treatment regimens against Gram-positive bacteria. We will extensively discuss these antibiotics under clinical development (phase I-III clinical trials) to combat Gram-positive bacteria, such as *Staphylococcus aureus*, *Enterococcus faecium* and *Streptococcus pneumoniae*. We will delve into the mechanism of actions, microbiological spectrum, and, where available, the pharmacokinetics, safety profile, and efficacy of these drugs, aiming to provide a comprehensive review to the involved stakeholders.

## 1. Introduction

Antimicrobial drugs have been the mainstay of treatment for bacterial infections for several decades [1]. The increased use of antibiotics worldwide in both human/veterinary medicine and agriculture has led to the emergence of resistant bacteria [2]. Infections caused by multiresistant (MDR) bacterial pathogens are associated with increased morbidity and mortality, as well as the excessive healthcare cost associated with the prevention and treatment of such infections that is estimated to amount to $20 billion in the United States and €1.6 billion in the European Union [1,3].

The increasing resistance rates to macrolides, fluoroquinolones, beta-lactams and other antibiotics commonly administered to combat Gram-positive bacteria are of great concern, especially in severe infections caused by MDR bacteria. Current treatments may soon be futile for previously treatable infections. During the last decade, 11 antibiotics with main activity against Gram-positive microorganisms have received international regulatory approval, i.e., ceftobiprole, ceftaroline, telavancin, oritavancin, dalbavancin, tedizolid, besifloxacin, delafloxacin, ozenoxacin, omadacycline and lefamulin. This review will delve into antimicrobial agents for the treatment of infections caused by MDR Gram-positive microorganisms currently undergoing clinical trials (Table 1; Table 2), including the mechanism of action, microbiological spectrum, safety and efficacy of these antibiotics. We will also briefly present alternative treatment approaches for Gram-positive microorganisms that are currently under clinical development. For novel, approved antibiotics against MDR Gram-positive microorganism, we refer the reader to other publications [4,5].

## 2. Phase III Drugs and Drugs with NDA Submitted

### 2.1. Dihydrofolate Reductase Inhibitors

#### Iclaprim

Iclaprim (trade name Mersarex), developed originally by Roche and currently under the ownership of Motif BioSciences Inc., was designed over 20 years ago with the intention of overcoming the mechanism of resistance against trimethoprim in staphylococcal species, especially *S. aureus* [6,7]. Specifically, iclaprim is a diaminopyrimidine with a 20-fold higher affinity to dihydrofolate reductase (DHFR) than trimethoprim, while maintaining the synergistic effect with sulfamethoxazole that is unique to DHFR inhibitors [8,9]. The in vitro spectrum of antibacterial activity of iclaprim covers many strains of drug-resistant *S. aureus*, including methicillin-resistant *S. aureus* (MRSA), vancomycin-intermediate and vancomycin-resistant, and the macrolide-, quinolone- and trimethoprim-resistant strains [8]. It also covers many strains of drug-resistant *Streptococcus pneumoniae*, including those that are resistant to penicillin, erythromycin, levofloxacin and trimethoprim/sulfamethoxazole [10,11]. In addition, iclaprim has antibacterial activity against vancomycin-resistant enterococci (VRE) strains [11].

Iclaprim can be administered orally and intravenously and was demonstrated to be effective and well-tolerated in complicated skin and skin structure infections (cSSSI), with a terminal elimination half-life of approximately three hours [12,13,14,15]. The distribution of iclaprim into the respiratory system is extensive, as its concentration exceeds MIC90 for Gram-positive respiratory bacteria by achieving 20 to 40 times greater concentration in the epithelial lining fluid (ELF) and alveolar macrophages (AM) than in plasma [12,16]. Iclaprim is not an inhibitor or inducer of CYP3A4 and CYP2C19 enzymes and is primarily excreted renally as conjugated metabolites [17]. No incidence of elevated serum creatinine was found in the phase 3 REVIVE trials, suggesting that there is no need to adjust the dose in renal impairment [17,18].

The main clinical indications that have undergone phase II or III clinical trials for iclaprim are acute bacterial skin and skin structure infections (ABSSSI), hospital-acquired bacterial pneumonia (HABP) and cSSSI [17,19]. A multi-centre, randomised, double-blind phase II study evaluating the efficacy and safety of intravenous iclaprim versus vancomycin for HABP (NCT00543608) demonstrated comparable clinical cure rates between iclaprim and vancomycin treatment groups, although the results were statistically non-significant as the study was prematurely terminated due to financial restraints on recruitment [20,21]. Mortality rates were also similar, without any new or unexpected treatment-emergent adverse events [21].

For the treatment of cSSSIs, a randomised, double-blind phase II trial found iclaprim to be noninferior to vancomycin for the endpoint of clinical cure [22]. In addition, two multi-centre, randomised, investigator-blind, phase III trials (ASSIST-1 and ASSIST 2, NCT00299520 and NCT00303550, respectively) found the clinical cure rate of iclaprim to be noninferior to linezolid [23,24]. Despite these clinical trials, both the U.S. Food and Drug Administration (FDA) and the European Medicines Agency (EMA) declined the approval of the use of iclaprim for cSSSIs in 2009, due to insufficient data demonstrating an acceptable margin of non-inferiority to the comparators, as well as concerns regarding QTc prolongation and liver toxicity [14,25]. To date, there have been no additional clinical trials for this clinical indication, as the focus for iclaprim has been transitioned to gain approval for ABSSSIs and HABP. In 2015, both Qualified Infectious Disease Product (QIDP) and Fast Track status have been granted to iclaprim for both indications [26].

REVIVE-1 and REVIVE-2, two randomised double-blind phase III clinical trials comparing the safety and efficacy of iclaprim versus vancomycin for ABSSSIs found that iclaprim 80 mg administered intravenously every 12 h is noninferior to vancomycin 15 mg/kg administered intravenously every 12 h for five to 14 days for achieving early clinical response within the first 48 to 72 h, as well as noninferiority at seven to 14 days after drug discontinuation (NCT02600611 and NCT02607618) [13,27]. Furthermore, pooled analysis of both trials showed that no patients from the iclaprim treatment group experienced clinically significant serum creatinine elevation, while two patients experienced nephrotoxicity related to vancomycin (0.7%) [28]. The commonest adverse drug reactions include headache (10.2%), nausea (9.9%), secondary ABSSSI (6.8%), and fatigue (6.1%) [27]. A total of 15 patients (5.5%) treated with iclaprim experienced increases in alanine aminotransferase or aspartate aminotransferase more than three times above the upper limit of normal, but these were reversible upon drug discontinuation [27]. Overall, the incidence of discontinuing iclaprim due to adverse drug reactions is lower than that of vancomycin (2.7% vs. 4.4%) [27].

In light of the results from these two clinical trials, the FDA accepted the new drug application (NDA) filing for iclaprim for ABSSSIs in August 2018, as well as granting iclaprim the QIDP designation [26,29]. However, in February 2019, the FDA asked for additional clinical data due to concerns over liver toxicity [30]. In addition to treating ABSSSIs, iclaprim is designated as an orphan drug for treating *Staphylococcus aureus* lung infections in patients with cystic fibrosis as of 2017 [31].

### 2.2. Ketolides

#### 2.2.1. Cethromycin

Cethromycin (trade name Restanza), developed by Advanced Life Sciences Holdings, Inc., is a second-generation ketolide, a subclass of macrolides that has a higher affinity for two binding sites (domain II and V) of the 23S ribosomal RNA [32]. Their mechanism of action allows ketolides to increase their activity against erythromycin-susceptible strains, while decreasing their susceptibility to efflux and methylation mechanisms in *S. pneumoniae* [32,33]. This allows cethromycin to have higher antibacterial activity than macrolides and telithromycin, the first ketolide approved in US that had two clinical indications withdrawn due to concerns of severe drug-induced hepatotoxicity [34,35]. Cethromycin has proven to be the most active agent against *S. pneumoniae* derived from community-acquired respiratory tract infections resistant to macrolides, followed by telithromycin, azithromycin, clarithromycin and erythromycin [36]. 

Cethromycin retains activity against telithromycin-resistant *S. pneumoniae*, possibly due to enhanced binding kinetics [37]. Moreover, cethromycin does not appear to cause visual disturbances, hepatotoxicity, and exacerbation of myasthenia gravis, which are all serious adverse effects associated with telithromycin [37]. Cethromycin is given orally once daily [37].

There have been two major phase III trials for evaluating the safety and efficacy of cethromycin, both of which were double-blind, randomised, parallel group, multi-centre, multinational clinical trials that compared cethromycin 300 mg once daily to clarithromycin 250 mg twice daily for the treatment of community-acquired bacterial pneumonia (CABP) in adults (NCT00336544, NCT00336505) [37]. Both studies showed that cethromycin is noninferior to clarithromycin for the treatment of mild to moderate community-acquired pneumonia (CAP) [37,38,39]. Common adverse drug reactions include nausea (2.69%), diarrhoea (5%), dysgeusia (11.15%), and headache (3.08%) [38].

To date, cethromycin has not been approved, despite its NDA being submitted in September 2008. According to the FDA, additional clinical data were needed to show its efficacy for CAP in moderate to severe CAP patients, although the FDA Anti-Infective Drugs Advisory Committee (AIDAC) has acknowledged the safety of cethromycin for the intent-to-treat population [40].

#### 2.2.2. Solithromycin

Solithromycin (trade name Solithera), developed by Cempra Pharmaceuticals, is a novel fluoroketolide antibiotic which inhibits bacterial translation by binding to the 50S ribosomal RNA, preventing protein synthesis [41]. The previously approved telithromycin is rarely used as its mechanism of action, which involves the blockade of nicotinic acetylcholine receptors, is known to cause serious side effects, including exacerbation of myasthenia gravis [42]. Solithromycin differs both chemically and biologically from telithromycin in its side chain and does not significantly block nicotinic acetylcholine receptors [42]. Solithromycin is active against MSSA, community-acquired MRSA strains and macrolide-resistant *Mycoplasma pneumoniae* [43].

In vitro, solithromycin was reported to be very potent against *S. pneumoniae* (MIC90 = 0.25 mg/L), and it was two- and ≥32-fold more active than telithromycin and clindamycin, respectively [44]. Solithromycin also demonstrated significantly greater potency than telithromycin, clarithromycin and azithromycin against intracellular *S. aureus*, in which the half maximal effective concentrations (EC50) of solithromycin compared to these drugs were three-, six- and 15-fold lower, respectively [45]. Phase I studies demonstrated that solithromycin, dosed at 400 mg orally once daily, resulted in excellent tissue distribution and high levels of accumulation in the lung against macrolide-resistant pneumococci [46]. Solithromycin has a MIC90 of 2 μg/mL for *E. faecalis*, two-fold lower than telithromycin (MIC90 = 4 μg/mL), and markedly more potent than erythromycin (MIC90 > 4 μg/mL) [43]. Studies have shown extensive penetration into the ELF in healthy subjects, evidenced by significantly higher steady-state concentrations in the ELF (2.4 to 28.6 times) than plasma concentrations after 400 mg of solithromycin dosed orally for five days [47]. The mean elimination half-life ranges from 3.16 to 7.42 h in a dose-dependent manner, and its oral bioavailability is unaffected by high-fat meals [47,48]. The elimination of solithromycin occurs primarily through hepatic transformation and excretion in the faeces [49]. However, in patients with hepatic dysfunction, clinical trials showed that solithromycin did not require dose adjustment [50]. Solithromycin was well tolerated and no significant differences in safety were found compared to healthy controls [50].

A multi-centre, randomised, double-blind phase III study investigating the efficacy and safety of oral solithromycin compared to moxifloxacin (NCT01756339), found that oral solithromycin was non-inferior to oral moxifloxacin for the treatment of CABP [51]. Another similar phase III study was conducted in patients ≥18 years of age with CABP to compare the safety and efficacy of intravenous/oral solithromycin to intravenous/oral moxifloxacin (NCT01968733) [52]. Both intravenous/oral solithromycin demonstrated non-inferiority to moxifloxacin [52]. The major adverse drug reactions include infusion site events (including infusion site pain, phlebitis, erythema, paresthesia, thrombosis, and infusion-related reactions) in 31.3% of patients [52]. Other common adverse drug reactions include gastrointestinal symptoms (diarrhoea 4.4%, nausea 3.2%; overall 12.5%), neurological symptoms (headache 3.5%, dizziness 2.5%; overall 6.7%) and hypokalemia (2.5%) [52]. Although adverse events were comparable between the two groups, mild and moderate infusion events led to a higher incidence of adverse events in the solithromycin group [52]. The safety and efficacy of solithromycin has also been investigated in adolescents and children with CABP (NCT02605122); however, this study was discontinued early due to a company business decision, and not related to safety and tolerability [53]. Another study, conducted in five chronic obstructive pulmonary disease (COPD) patients to examine the effect of solithromycin as an anti-inflammatory treatment for COPD (NCT02628769), was terminated early due to cholestatic hepatitis in one subject and alanine aminotransaminase (ALT) elevation in two others after the administration of solithromycin [54].

Solithromycin has substantial in vitro activity against *Neisseria gonorrhoeae*. A phase III, open-label, multi-centre trial (SOLITAIRE-U) evaluated a single 1000 mg dose of solithromycin versus ceftriaxone plus azithromycin for the treatment of uncomplicated gonorrhoea in 262 patients [55]. Solithromycin eradicated 92% of all microbiologically evaluable cases of genital gonorrhoea in one week [55]. However, it failed to reach the non-inferiority margin of 10% compared with the control treatment [55].

An application was submitted for the approval of solithromycin to the FDA [56]. However, in December 2016, the Complete Response Letter from the FDA noted that additional clinical safety information and manufacturing facility inspection deficiencies would need to be resolved before the drug application can be approved [41,56]. The FDA agreed that solithromycin was an effective treatment for CABP but stated that the risk of hepatotoxicity was not adequately characterised, due to the small sample size of 920 patients [41,56]. It was recommended that a comparative study be conducted on approximately 9000 patients to exclude drug-induced liver injury events [41,56].

### 2.3. Oxazolidinones

#### Contezolid

Contezolid (formerly known as MRX-1), developed by MicuRx Pharmaceuticals, Inc., is a new oxazolidinone with the same core structure as linezolid [57]. Contezolid and its prodrug contezolid acefosamil (MRX-4) were granted QIDP classification and fast-track status by the FDA in September 2018 for the treatment of ABSSSIs [58].

Similar to linezolid, the mechanism of action of contezolid involves bacterial protein synthesis inhibition by binding to the ribosomal RNA [52,59]. In vitro, contezolid has potent activity against Gram-positive pathogens, such as MRSA, penicillin-resistant *S. pneumoniae*, penicillin-intermediate *S. pneumoniae*, and VRE [57]. Against both drug-susceptible and drug-resistant *Mycobacterium tuberculosis* (TB), the in vitro activity of contezolid is similar to that of linezolid [60]. Orally administered contezolid has been shown to have the same or better efficacy in systemic and local infection mouse models [57]. Against both drug-susceptible and drug-resistant TB, the in vivo activity and in vitro activity of contezolid in a murine tuberculosis model was also comparable to that of linezolid [60].

Contezolid has a mean elimination half-life of 2.2 to 4.9 h in a dose-dependent manner (2.2 h with 300 mg, 4.9 h with 900 mg), and its oral bioavailability is enhanced with fat-containing meals [61].

Phase I clinical trials demonstrated that contezolid had decreased haematological toxicity compared to linezolid and had the potential to improve the ease of use in patients with drug-resistant TB [61,62]. Hematological markers such as platelets, neutrophils, red blood cells, and reticulocytes were all unchanged at up to 800 mg oral doses in two phase I trials [61,63]. In addition, there is a significantly lower risk of drug–drug interactions with monoamine oxidase inhibitors (MAOi) compared to linezolid [61,63]. Mild alanine transaminase (ALT) elevations were observed in a phase I trial (60%, *n* = 10); all of these patients’ ALT levels returned to normal in the follow-up visit of the trial [63]. No other liver function tests were elevated [63]. In the same phase I trial, headache (10%), lethargy (10%), and blurred vision (10%) were also reported by one patient each, but none were rated as severe [63]. A phase II trial (NCT02269319) has been successfully completed. Contezolid was also evaluated in a double-blind, phase III clinical trial at 50 sites in China for the treatment of cSSSIs [64]. This pivotal study reportedly found contezolid to meet the primary endpoint of noninferiority (93.0%) compared to linezolid (93.4%) for the clinical cure rate [65]. Contezolid was also associated with fewer drug-related haematological adverse events [65]. Of the patients that received more than 10 days of therapy, 2.5% of contezolid patients experienced a platelet count reduction of more than 30%, compared with 25.4% of linezolid patients [65]. In light of these results, MicuRx Pharmaceuticals Inc. recently reported that it will be preparing to file an NDA for contezolid with China’s National Medical Products Administration [66].

Contezolid acefosamil (formerly known as MRX-4), the water-soluble prodrug of contezolid, is currently being tested in clinical trials to determine the efficacy of its intravenous form, highly desirable for the treatment of serious MRSA and VRE infections in hospital, and the enhanced oral form for its potential in outpatient treatment [58]. Phase I studies of the intravenous form (NCT03033329) and the enhanced oral form (NCT03033342) that evaluated the safety, tolerability and pharmacokinetics of contezolid acefosamil have been completed in healthy participants [67,68]. A phase II trial (NCT03747497) is currently underway comparing the safety and efficacy profiles of contezolid acefosamil and linezolid both intravenously and orally in subjects with ABSSSI in the United States [69]. According to preliminary company report, the findings of this trial support the non-inferiority of contezolid compared with linezolid for this indication [70]. MicuRx Pharmaceuticals plans to initiate phase III trials for contezolid acefosamil in skin and soft tissue in China and USA in 2020 [71].

### 2.4. Quinolones

#### 2.4.1. Lascufloxacin

Lascufloxacin, developed by Kyorin Pharmaceutical Co., Ltd., is a new fluoroquinolone developed in Japan for CAP and other respiratory tract infections, with a similar mechanism of action to other fluoroquinolones, by binding to DNA gyrase (subunits GyrA and GyrB) and topoisomerase IV (subunits parC and ParE) to inhibit DNA synthesis [72].

Its spectrum of activity is similar to levofloxacin, demonstrating the most potent activity against Gram-positive bacteria among the quinolones and incomplete cross-resistance against existing quinolone-resistant strains. The MIC90 against MRSA was 2 μg/mL, almost the same as linezolid and vancomycin, and 32 to >64 times higher than levofloxacin, garenoxacin and ciprofloxacin [73]. It is available in oral and intravenous formulations [72]. An in vitro comparison study showed that lascufloxacin had the lowest MICs against Gram-positive pathogens such as methicillin-susceptible *S. aureus* (MSSA), MRSA, *S. epidermidis, E. faecalis, S. pyogenes, S. agalactiae,* and penicillin-susceptible and penicillin-resistant *S. pneumoniae* among quinolones [73]. Lascufloxacin was eliminated with an average half-life of 16.1 h when given at 100 mg orally in healthy subjects [73]. Furthermore, a pharmacological study has indicated extensive distribution into the lungs with ELF, with a plasma concentration ranging from 15.0 to 22.4 [74]. Lascufloxacin was well-tolerated with no serious adverse events. The most common adverse event was increased C-reactive protein, seen in nine patients (9/31), fever in six participants (6/31), leukocytosis (3/31) and headache (3/31) in three subjects each, all of which were considered related to the bronchoalveolar lavage procedure, and not the study drug [74].

In 2017, there were five phase III trials registered in the Japanese clinical trial registry, and in 2018 an NDA was filed in Japan [75,76]. It was approved in September 2019 in Japan for community-acquired pneumonia and otorhinolaryngological infections [77].

#### 2.4.2. Nemonoxacin

Nemonoxacin (trade name Taigexyn), developed by TaiGen Biotechnology Co., Ltd., is a novel non-fluorinated quinolone with potent in vitro activity against Gram-positive bacteria, especially multidrug-resistant *S. pneumoniae*, MRSA and vancomycin-resistant pathogens [78]. Oral nemonoxacin rapidly reaches maximum concentration one to two hours in the fasting state and has a long half-life of more than 10 h [78]. Approximately 60–75% is excreted via the kidneys over 24 to 72 h [78]. The addition of a methoxy group at the C-8 position allows nemonoxacin to target both topoisomerase IV and II, resulting in an improved spectrum of activity, whilst the removal of the fluorine residue is thought to reduce the incidence of toxic side effects [79,80]. Nemonoxacin has a lower propensity for selecting resistant pathogens than fluoroquinolones, as bacteria only become resistant when three different mutations occur in their quinolone resistance-determining regions [78].

A multi-centre, double-blind, randomised controlled phase III trial (NCT01529476) was conducted to assess the efficacy and safety of oral nemonoxacin compared with levofloxacin, which is the current recommendation for the treatment of CAP in adult patients [81]. Nemonoxacin was found to have a microbiological success rate that was not inferior to that of levofloxacin (92.1% vs 91.7%) for *S. pneumoniae* and *S. aureus* as well as good efficacy against atypical CAP pathogens, *Mycoplasma pneumoniae, Chlamydophilia pneumoniae* and *Legionella pneumophila* [81]. The common adverse drug reactions include nausea (3.4%), dizziness (2.8%), abdominal discomfort (2.2%) and vomiting (1.7%). Laboratories have found abnormalities including elevated alanine aminotransferase (5.9%), aspartate aminotransferase (2.5%), and leukopenia (2%) [81]. The drug-related clinical adverse events were mainly ALT elevation, decreased white blood cell (WBC) count and nausea, all of which were mild to moderate severity [81]. Overall, nemonoxacin was found to be as effective and safe as levofloxacin in terms of clinical cure rates, and safety profile, making it a suitable alternative to fluoroquinolones for treating adult CAP patients [81]. In a phase II trial assessing the safety and efficacy of nemonoxacin in patients with diabetic foot infections (NCT00685698), some serious adverse events were noted, such as gangrene, abscess limb, osteomyelitis and increased blood glucose and blood pressure [82]. Currently, a phase I study is investigating the use of nemonoxacin malate capsules in subjects with severe impaired renal function (NCT02840812) [83].

Oral nemonoxacin is approved in Taiwan and China for the treatment of CAP in adults [81]. TaiGen Biotechnology has also been granted both QIDP and fast-track designations by the FDA for CAP and ABSSSIs [84].

#### 2.4.3. Levonadifloxacin

Levonadifloxacin (WCK771) and its oral form prodrug alalevonadifloxacin (WCK2349), developed by Wockhardt Ltd., are broad-spectrum fluoroquinolones developed to combat MRSA and quinolone-resistant staphylococci and other drug-resistant bacteria for the treatment of ABSSSIs and HABP [85]. Levonadifloxacin inhibits bacterial DNA gyrase in addition to topoisomerase IV which classical quinolones also targets, leading to a high potency against MRSA and staphylococci resistant to levofloxacin and moxifloxacin [86]. In vitro studies have also shown the bactericidal effect of levonadifloxacin on biofilm-embedded quinolone-resistant *S. aureus* and MRSA [87]. The NorA efflux pump does not affect the activity of Levonadifloxacin, demonstrating a significant advantage over other quinolones, including ciprofloxacin, norfloxacin, clinafloxacin and gemifloxacin, which express efflux-mediated fluoroquinolone resistance [88].

In vitro, levonadifloxacin was highly potent against quinolone-susceptible staphylococci (MIC90 = 0.015 μg/mL) and quinolone-resistant isolates (MIC90 = 1 μg/mL) [88]. In comparison, other quinolones had MIC90 >4 μg/mL when tested against quinolone-resistant staphylococci [88]. In vivo studies demonstrated oral levonadifloxacin to have superior efficacy against systemic MSSA infections when compared to sparfloxacin and moxifloxacin [89]. Against six clinical isolates of quinolone-resistant MRSA, the efficacy of levonadifloxacin was significantly superior to those of trovafloxacin and sparfloxacin for five of the strains [89]. Levonadifloxacin was found to be the most potent agent when tested against 234 clinical isolates of MSSA and MSSE, and possessed a potency comparable to clinafloxacin, one of the most potent anti-MRSA quinolones, with a 10-fold higher effective dose [89]. When four doses of 50 mg/kg were administered subcutaneously, MRSA was eradicated from mouse liver, spleen, kidney and lungs when administered subcutaneously [89].

A phase I, multiple-dose study showed that following the oral administration of levonadifloxacin (pro-drug WCK2349) in healthy adult human subjects, the penetration ratios for ELF and AM to plasma concentration were 7.66 and 1.58, respectively, supporting its use for lower respiratory tract infections (NCT02253342) [90]. The elimination half-life, clearance and volume of distribution for oral levonadifloxacin were reported to be 6.35 h, 8.17 L/hour and 59.2 L, respectively [90]. Out of 31 subjects, 12 (38.7%) developed mild adverse events, including photophobia (four out of 12), dysgeusia (four out of 12), leukopenia, back pain, headache and skin papule. No severe adverse events nor clinically significant changes in physical examination findings, vital signs or electrocardiograms were observed [90].

In September 2014, levonadifloxacin received QIDP status from the FDA [91]. In 2016, two trials were conducted in healthy volunteers in the United States to investigate the safety and tolerability of multiple twice-daily doses of levonadifloxacin [92]. Multiple escalating doses of levonadifloxacin were well tolerated, with no serious adverse events, clinical abnormalities or deaths reported [92]. One subject developed the moderate adverse event of hypersensitivity—however, this resolved by the end of the study [92]. In November 2018, a phase III interventional clinical trial (NCT03405064) was completed in India comparing 800 mg levonadifloxacin intravenously twice daily with 600 mg linezolid intravenously twice daily for the treatment of ABSSSIs, but the findings of this study have not yet been published [93].

#### 2.4.4. Zabofloxacin

Zabofloxacin (DW-224a), synthesized by Dong Wha Pharmaceutical Industry Ltd., is a novel fluoroquinolone antibiotic which inhibits bacterial DNA gyrase and topoisomerase IV [94]. It has demonstrated more potency against Gram-positive bacteria when compared with other fluoroquinolones including ciprofloxacin, moxifloxacin and gemofloxacin [95]. It has a wide antibacterial spectrum against Gram-positive pathogens including MRSA, methicillin-resistant coagulase-negative staphylococci, *S. pyogenes, E. faecalis* and especially *S. pneumoniae,* which is most frequently associated with CABP [95].

In the first phase I study investigating the pharmacokinetics of a single dose of either a 400 mg zabofloxacin hydrochloride or a 488 mg zabofloxacin aspartate capsule in 32 healthy male participants, its half-life was 8.24–8.32 h after reaching maximum concentration within 1 to 2 h following oral administration to 29 healthy volunteers (NCT01341249) [96]. Both zabofloxacin tablets were well tolerated, and all adverse events were transient and mild or moderate, and not related to the drug [96]. They included nausea which occurred in two subjects (7%), and presyncope (3%), hypotension (3%) and somnolence (3%) reported in one subject each [96].

In a multi-centre, double-blind, non-inferiority, randomised controlled phase III trial (NCT01658020), zabofloxacin was shown to be non-inferior to moxifloxacin, with better patient-oriented outcomes in treating infections in patients with COPD exacerbations [97]. It was approved by the Ministry of Food and Drug Safety of Korea in 2015 for the treatment of acute exacerbations of COPD, and Dong Wha Pharmaceutical Industry also obtained approval from the FDA for its phase III clinical trial involving CABP. The company plans to expand the scope of its use to include the treatment of urinary tract infections [98]. A multi-centre, double-blind, randomised controlled phase II trial (NCT01081964) evaluating the safety and efficacy of oral zabofloxacin in CABP started its recruitment in 2010 in the United States; however, it was terminated prematurely in 2012 due to financial considerations [99].

### 2.5. Defensin Mimetic

#### Brilacidin

Brilacidin, previously known as PMX30063, is a novel defensin mimetic developed by Innovation Pharmaceuticals Inc. that is being evaluated as an ocular anti-infective [100]. Brilacidin mimics naturally occurring defensin, which serves as a first line of defence against microbes on the ocular surface [100,101]. Its unique mechanism of action may decrease the risk of antibiotic resistance. In vitro, brilacidin was more potent against Gram-positive bacteria than Gram-negative, with both *S. aureus* and *S. epidermidis* possessing the lowest MIC90s (0.25 μg/mL) among the bacterial groups tested [100]. A phase IIa clinical trial was successfully conducted in 2010 investigating the safety and efficacy of brilacidin in patients with ABSSSI (NCT01211470) [102,103]. The results were positive, with three-day cure rates for all dosing regimens, comparable with seven days of daptomycin, thus indicating the potential for shorter dosing regimens and reduced complications, risk of antibiotic resistance and healthcare cost [104]. Treatment-related serious adverse events included one incidence of hypertension in the medium- and high-dose regimens, and one of increased platelets in the low-dose regimen [104]. A randomised, multi-centre, phase IIb clinical trial was completed in 2014, demonstrating that a single dose of Brilacidin was as effective as a seven-day dosing regimen of daptomycin in the treatment of ABSSSI (NCT02052388) [105]. Six serious adverse events were reported; however, none were considered to be related to the study drug [105]. Brilacidin is currently being advanced into phase III clinical trials after USFDA granted QIDP in 2014 for the treatment of ABSSSI [106].

## 3. Phase II Drugs

### 3.1. β-lactams

#### 3.1.1. Razupenem

Razupenem (previously known as PTZ601, PZ-601, SMP-601 or SM-216601), owned by Novartis who acquired PZ-601 in a merger deal with Protez Pharameuticals, is a broad-spectrum injectable antibiotic from the carbapenem subgroup of beta-lactam antibiotics, which acts by inhibiting peptidoglycan biosynthesis in the bacterial cell wall [107,108].

In vitro, razupenem demonstrated a broad-spectrum activity against various aerobic bacteria, including MRSA, penicillin-resistant *S. pneumoniae,* vancomycin-resistant *E. faecium* and ampicillin-resistant *H. influenzae* [109]. In mouse models, the therapeutic efficacy of razupenem reflected its in vitro activity [109,110]. Another in vitro pharmacokinetic model was used to simulate serum drug concentrations of razupenem in humans with administration of 1g intravenously every 12 h [110]. The half-life of razupenem after a one-hour infusion with dosing every 12 h for 48 h was found to be 1.5 h [110].

A multi-centre, randomised phase II clinical trial was conducted to evaluate the safety, efficacy and pharmacokinetics of razupenem in the treatment of complicated skin and skin structure infections (NCT00671580) [111]. Novartis has recently abandoned further development of PZ-601 due to high rates of rash adverse events in phase II clinical trials [112].

#### 3.1.2. Tomopenem

Tomopenem (formerly CS-023), developed by Daiichi Sankyo Research Laboratories, is a carbapenem beta-lactam antibiotic, with a broad-spectrum coverage of both Gram-positive and Gram-negative pathogens [113]. It has unique antibacterial activity against MRSA, unlike the currently available carbapenems, imipenem and meropenem, which are ineffective against MRSA [113]. Tomopenem inhibits the activity of penicillin-binding proteins and disrupts the biosynthesis of peptidoglycans in the bacterial cell wall [114]. As with other new carbapenems, tomopenem as a low propensity for the emergence of resistance [115].

In vitro studies of 60 German clinical isolates of MRSA showed tomopenem to have significantly lower MIC90 (8 μg/mL) than imipenem (>32 μg/mL) and meropenem (32 μg/mL) [113]. This is supported by another in vivo study in which tomopenem showed highly potent activity against MSSA and methicillin-susceptible *S. epidermidis* [116]. More significantly, tomopenem had a MIC90 (8 μg/mL) that was four-fold lower than imipenem and meropenem against MRSA. Although there was potent activity against *E. faecalis*, imipenem exhibited higher activity than tomopenem [116]. The inhibitory concentration value of tomopenem was also more than 15-fold lower than that of imipenem and meropenem [113]. In murine models of MRSA infection, tomopenem showed bactericidal effects against all nine strains of MRSA with MICs of ≤16 μg/mL [117]. The half-life of tomopenem was 0.197 h in sera and 0.343 h in the lungs and the percentage of the dosage interval in which the serum level exceeded the MIC (%T > MIC) for tomopenem was 16% in sera and 15% in the lungs [118]. Tomopenem exhibited a half-life of 1.7 h after administration in healthy volunteers, twice longer than imipenem or meropenem, making it more advantageous as it allows for more convenient dosing intervals [119].

As carbapenems are primarily excreted by the kidneys, a clinical trial was conducted to determine the effect of renal impairment on tomopenem [114]. Tomopenem was given as a 1500mg intravenous infusion over 60mins and showed a significant effect on the pharmacokinetics and elimination of the drug [114]. The half-life of tomopenem decreased with the increasing impairment of renal function; normal renal function (t_1/2_ = 2.23 h), mild renal impairment (t_1/2_ = 3.00 h), moderate (t_1/2_ = 4.59 h) and severely (t_1/2_ = 7.94 h) impaired renal function [114]. Adverse events were experienced by 18 subjects (56%), with the majority being dizziness, dyspepsia and flatulence [114]. Multiple phase II clinical trials in USA and Europe for Gram-negative and Gram-positive infections were discontinued in 2008 due to financial resource limitations [120].

### 3.2. Brilacidin

#### Radezolid

Radezolid (formerly RX-1741), developed by Melinta Therapeutics, Inc., is a novel oxazolidinone antibiotic that shares many similarities with tedizolid [121]. Oxazolidinones bind to the P site of the ribosomal 50S subunit and inhibit protein synthesis [122]. Radezolid poses a biaryl spacer and a heteroaryl side chain, which allows for increased ionisation and hydrophilicity at physiological pH, therefore giving it an advantage over current drugs in the class [123]. In vitro, radezolid is two times more active against *S. aureus* and four to 16 times more potent against *S. pneumoniae* and enterococci than linezolid [108].

A multi-centre, randomised phase II clinical trial was conducted to compare the safety and efficacy of oral radezolid to linezolid in the treatment of uncomplicated skin infections in adult patients (NCT00646958) [124]. This study demonstrated promising activity against several pathogens as well as similar clinical cure rates compared to linezolid [125]. Compared to linezolid, radezolid had increased potency towards intra-phagosomal *S. aureus* and its activity was unaffected by resistance to linezolid [126]. Radezolid was well tolerated, and the most common adverse events reported were gastrointestinal symptoms [125]. Another multi-centre randomised double-blind phase II study was conducted to evaluate the safety and efficacy of radezolid in 158 adult patients with community-acquired pneumonia (NCT00640926) [127]. Adverse effects included diarrhoea (35/158), fungal infections (5/158), pneumonia (6/158), dizziness (4/158) and headache (6/158) [127]. Serious adverse events were also reported, including *Pneumocystis jirovecii* infection (1/53, 1.89%), abnormal hepatic enzymes (1/53, 1.89%), dehydration (1/53, 1.89%), pleural effusion (1/52, 1.92%), acute renal failure (1/52, 1.92%), diabetes mellitus (1/53, 1.89%) and adenocarcinoma of the lung (1/53, 1.89%) [127].

Currently, no phase III clinicals have been planned. Radezolid was granted a QIDP in 2018 for the indication of bacterial vaginosis after it demonstrated in vitro activity against bacteria usually associated with bacterial vaginosis [128].

### 3.3. Novel Bacterial Topoisomerase II Inhibitor

#### Gepotidacin

Gepotidacin (formerly GSK2140944), developed by GlaxoSmithKline, is a triazaacenaphthylene antibacterial agent that is also the first in a new antibacterial drug class called novel bacterial topoisomerase II inhibitor (NBTI) [129]. Specifically, its mechanism of action involves a unique binding mode that allows it to bypass fluoroquinolone resistance, as demonstrated by its in vitro antibacterial activity against MRSA and levofloxacin-resistant (FQR) and MDR *S. aureus* [129,130].

In addition, the spectrum of in vitro antibacterial activity of gepotidacin covers both ciprofloxacin-susceptible and -resistant strains of *Neisseria gonorrhoeae* [131,132]. Murine infection models have demonstrated gepotidacin to have a short half-life, with q24 h dosing intervals proving inadequate, resulting in regrowth within the murine model [133]. Repeat dosing will only lead to Cmax increases of 9–18%, due to the short half-life of the drug [134]. However, gepotidacin has the potential for drug–drug interactions as it is a CYP3A4 substrate; therefore, plasma concentrations may be higher in patients with impaired clearance of the drug [134].

A non-randomised, two-period, cross-over study evaluated the pharmacokinetics, metabolism and excretion of gepotidacin after 1000 mg intravenous and 2000 mg oral dose administration containing radioactive doses, in six healthy male subjects (NCT02000765) [135]. Urinary elimination (59%) was predominant in the intravenous route due to the relative polar nature of gepotidacin, whereas faecal elimination (53%) was predominant in the oral route due to high levels of unabsorbed drug [135]. Gepotidacin is readily eliminated from plasma with a half-life of 12.1–12.6 h [135]. Gepotidacin can be given intravenously or orally, as its absolute oral bioavailability is approximately 50% [135]. Flatulence (6/6, 100%) was the most commonly reported adverse event in the intravenous group, whereas the most common adverse event in the oral group was diarrhoea (4/6, 67%), both of which were deemed to be related to gepotidacin by the investigator [135]. Other studies have reported that the commonest adverse effects are nausea (20%) and diarrhoea (13%), both of which increase in incidence with higher dosages [136]. A phase I trial that investigated the adverse effects of gepotidacin on cardiac conduction in healthy volunteers (NCT02257398) found that infusion at 1000 mg and 1800 mg over two hours can lead to QTc prolongation by 12 ms and 22 ms, respectively [134].

Two phase II dose-ranging studies were completed for the evaluation of the efficacy, safety and tolerability of gepotidacin for acute bacterial skin and skin structure infections (ABSSSIs) and uncomplicated urogenital gonorrhoea (NCT02045797 and NCT02294682, respectively) [137,138]. The first study consisted of a randomised, two-part, multi-centre clinical trial that found both intravenous and oral gepotidacin to be safe and effective for patients with suspected or confirmed Gram-positive ABSSSIs, with the highest clinical success rate achieved by gepotidacin 1000 mg every eight hours [136]. The second study consisted of a randomised, multi-centre clinical trial that found single oral doses of gepotidacin at either 1500 mg or 3000 mg are over 95% effective for eradicating N. gonorrhoeae in uncomplicated urogenital gonorrhoea [139]. This study showed no treatment-limiting adverse effects for either dose [139]. Similarly to previous reports, the most frequently reported adverse events were diarrhoea (27%), flatulence (23%), abdominal pain (15%), and nausea (13%) [139].

As of July 2019, no further clinical trials have been conducted for the treatment of ABSSSIs; however, a phase III, randomised, multi-centre clinical trial (NCT04010539) evaluating the efficacy and safety of oral gepotidacin in comparison to intramuscular ceftriaxone plus azithromycin for the treatment of uncomplicated urogenital gonorrhoea in approximately 600 adolescents and adults, is currently recruiting [140]. Gepotidacin will also be further investigated for uncomplicated urinary tract infections in a phase III trial [141].

### 3.4. FabI Inhibitor

#### 3.4.1. DEBIO1450

Debio1450 (previously AFN-1720), the prodrug of Debio1452 (previously AFN-1252), developed by Debiopharm, is an investigational antimicrobial agent that acts by inhibiting FabI, an enzyme necessary for fatty acid biosynthesis in staphylococci [142]. This anti-staphylococcal activity may prove beneficial for use in skin and skin structures [143]. Debio1452 has demonstrated a lack of activity against streptococci, enterococci and non-fermentative Gram-negative bacteria, reducing the effect on normal bacterial flora and adverse events [144]. This unique mechanism of action is not compromised by interactions with major antibiotic classes, reducing the risk of cross-infection with other antibiotics [142].

Patient isolates from 35 countries were tested for susceptibility to Debio1452 and 10 comparators including erythromycin, levofloxacin, clindamycin and trimethoprim-sulfamethoxazole [145]. Debio1452 demonstrated significantly greater activity overall (MIC50 0.004 μg/mL) compared to the comparators, as well as potent activity against MSSA and MRSA and strains resistant to current antimicrobial agents [145]. In addition, Debio1452 activity is not affected by the presence of lung surfactants, unlike daptomycin [142]. In vitro studies have shown that Debio1452 is well-absorbed and has solubility-limited absorption [143]. In a mouse model of septicaemia, Debio1452 demonstrated 100% protection from a potentially lethal infection of *S. aureus* [146]. A microdosing study of Debio-1452 in healthy volunteers demonstrated similar pharmacokinetics in intravenous and oral administration [143]. It has a long terminal half-life of approximately seven hours and 83% bioavailability, demonstrating minimal first-pass metabolism [143]. Debio1252 is primarily excreted via the urinary and faecal routes in both intravenous and oral administration [143].

A phase II multi-centre study was completed in 2012 to evaluate the efficacy, safety and tolerability of an oral daily 400 mg dose of Debio1452 in 103 participants with ABSSSI due to staphylococci, many with significant comorbidities, including intravenous drug use, human immunodeficiency virus infection, hepatitis infection and tuberculosis [147,148] (NCT01519492). The microbiological eradication rate was 93.2% at short-term follow up and 91.9% at long-term follow-up, including MRSA (91.9%) and MSSA (92.3%) [147]. Drug-related adverse events were experienced by 69 (67.0%) patients, with the most common being headache (26.2%), nausea (21.4%), vomiting (7.8%), skin infection (6.8%) and pruritis (5.8%) [147]. Shifts in liver function tests were uncommon and generally associated with those with hepatitis C virus infection and drug or alcohol abuse [147].

A phase I study studying the pharmacokinetics of Debio1450 in healthy adult volunteers was completed in 2015; however, no results have been posted [149]. Another phase II study was conducted to evaluate the efficacy of two doses of intravenous and oral Debio1450 compared to intravenous vancomycin and oral linezolid for the treatment of ABSSSI caused by staphylococcal infections (NCT02426918) [150]. Two (1.82%) subjects given 80 mg/120 mg twice daily of Debio1450 experienced skin infections and one (0.93%) subject experienced an overdose when given 160 mg/240 mg twice daily dose, compared to one (0.93%) subject given vancomycin/linezolid twice daily experiencing cellulitis [150]. Other adverse events were also reported, including headache in 10 subjects (9.09%) when given 80 mg/120 mg twice daily of Debio1450, and 18 (16.82%) when given 160 mg/240 mg twice daily, compared to nine (8.41%) subjects in the vancomycin/linezolid group [150].

#### 3.4.2. CG400549

CG400549, developed by CrystalGenomics Inc., is another FabI inhibitor, developed to combat resistant bacterial strains including MRSA and VRSA [151]. In vitro, clinical isolates of S. aureus were tested for susceptibility against CG400549 and comparators including erythromycin, ciprofloxacin, sparfloxacin, linezolid and vancomycin [152]. It was found to have potent in vitro antibacterial activity against staphylococci and was four to eight times more active than vancomycin and linezolid [152]. The MIC90s of CG400549 for MSSA and MRSA were 0.25 mg/L, the lowest amongst the 10 tested compounds [152]. The in vivo activity in mice models demonstrated similar in vivo efficacy against systemic infections caused by antibiotic-resistant strains such as MRSA, methicillin-resistant but quinolone-susceptible, and both methicillin-resistant and quinolone-resistant strains, with ED50 ranging from 5.12 to 10.36 for the oral route and 25.93 to 34.45 mg/kg for the subcutaneous route [152]. CG400549 was found to be equally as active against MSSA and MRSA, but showed no activity against streptococci, enterococci and Gram-negative bacteria [152]. In 2013, positive data were reported by CrystalGenomics from a phase IIa study evaluating the safety, pharmacokinetics and efficacy of 960 mg of CG400549 orally administered once daily in participants with complicated ABSSSI caused by MRSA (NCT01593761) [153,154]. At the early clinical evaluation point, 90.9% of subjects were considered stable or improving, with 100% test of cure at Day 21 to 28 [153]. No deaths, serious adverse events or discontinuations were reported and most adverse events were unrelated to the study drug [153]. However, although formal results have been submitted, they have not yet been published.

## 4. Phase I Drugs

### 4.1. Aminoglycosides

#### ME1100

ME1100 (trade name Habekacin), currently under development by Meiji Seika Pharma Co., Ltd., is a specialised inhalation solution of arbekacin, a broad-spectrum aminoglycoside licensed for systemic use in Japan since 1990 [155]. Its mechanism of action involves binding to 50S and 30S ribosomal subunits to inhibit bacterial protein synthesis [156]. Under the trade name Habekacin, arbekacin has been used clinically for sepsis and pneumonia caused by MRSA, as it demonstrates stability in the presence of aminoglycoside-modifying enzymes in MRSA [157]. In a large surveillance study in the United States, arbekacin was found to be very active against MRSA, *S. aureus* with heterogeneous resistance to vancomycin, community-acquired MRSA, and gentamicin-resistant *S. aureus* [158]. In addition, arbekacin has remained highly bactericidal against a wide range of hospital-acquired and ventilator-associated bacterial pathogens including both Gram-positive and Gram-negative bacteria, *Pseudomonas aeruginosa, S. pneumoniae, H. influenzae, Acinetobacter baumannii*, and Enterobacteriaceae species [158].

As of 2015, ME1100 qualified for QIDP status for the adjunctive treatment of mechanically ventilated patients with bacterial pneumonia [159]. To date, there have been three phase I clinical studies that were completed examining the pharmacokinetic properties and safety profile of ME1100 in healthy volunteers (NCT01907776 and NCT01961830) and patients on mechanical ventilation (NCT02459158) [160,161]. A population pharmacokinetic model was subsequently developed from the results of these studies [162]. Nephrotoxicity is a major drug-related adverse effect related to arbekacin use and has been associated with both arkebacin trough concentrations and total cumulative doses [162]. This model estimates that approximately 19.5% of the inhaled dose of ME1100 reaches the systemic circulation, similar to inhaled amikacin [162,163]. Of the subjects that received inhaled ME1100, 59/66 had a quantifiable ELF concentration [162]. This formulation allows for the delivery of ME1100 directly to the lungs, rather than via penetration from the systemic circulation, allowing for optimal exposures for pneumonia patients whilst minimising the systemic effects [162].

### 4.2. Quinolones

#### 4.2.1. Alalevonadifloxacin

Alalevonadifloxacin (WCK2349), developed by Wockhardt Ltd., is an L-alanine ester prodrug of levonadifloxacin (WCK771) discussed above, the arginine salt of S-(-)-nadifloxacin that has a greater in vitro potency than nadifloxacin [164,165]. Its mechanism involves a high affinity to staphylococcal DNA gyrase, which allows for consistently effective activity against quinolone-resistant *S. aureus* [87]. Alalevonadifloxacin is highly water-soluble and has a high oral bioavailability of approximately 89%, making it a suitable candidate for oral administration [90,164,165]. The available studies demonstrated that its in vitro antibacterial spectrum of activity covers fluoroquinolone-resistant strains of MRSA, in addition to common respiratory pathogens such as *S. pneumoniae, H. influenzae*, and *M. catarrhalis* [166,167]. Both levo- and alalevonadifloxacin are being developed for the clinical indications of ABSSSIs, CABP and HABP caused by MRSA [90]. 

Alalevonadifloxacin has completed a phase III clinical trial in India for the treatment of ABSSSIs [168]. Meanwhile, in the United States, the only registered clinical trials are phase I pharmacokinetic studies (NCT02253342, NCT02244827, NCT01875939, NCT02217930) [160,169,170]. A multiple-dose, open-label phase I study (NCT02253342) found that alalevonadifloxacin at 1000mg twice daily for five days is safe and well-tolerated in healthy adults [90]. 

#### 4.2.2. Avarofloxacin

Avarofloxacin (also known as JNJ-Q2 or acorafloxacin), discovered by Johnson and Johnson, developed by Furiex Pharmaceuticals and currently licensed to Allergan plc., is a fifth-generation aminoethylidenylpiperidine fluoroquinolone [171]. Though its mechanism of action is similar to other fluoroquinolones, avarofloxacin exhibits a more balanced affinity for both DNA gyrase and DNA topoisomerase IV, which is attributed to the addition of a methoxyl group at the C-8 position of the fluoroquinolone nucleus [172]. Its spectrum of in vitro and in vivo bactericidal activity covers a wide range of Gram-positive and Gram-negative bacteria, among which include fluoroquinolone-resistant MRSA and fluoroquinolone-resistant *S. pneumoniae* [173,174]. Multiple in vitro studies also found that avarofloxacin has a relatively lower risk for developing resistant mechanisms in *S. pneumoniae* and *S. aureus* than other fluoroquinolones [86,174,175]. In terms of pharmacokinetic parameters, avarofloxacin has an estimated absolute oral bioavailability of 65%, and is extensively distributed into ELF and AM [176]. However, only 12% of active drug is excreted by urine, which suggests that its clinical utility for treating urinary tract infections may be limited [171,176]. The half-life of avarofloxacin is 13 to 20 h and is renally cleared at a rate of 0.58 L/h [176]. Data from studies evaluating the safety of avarofloxacin showed that it was well tolerated up to 150 mg twice daily intravenously. Across the multiple doses, seven adverse events, namely nausea (1/18), vomiting (1/18), diarrhoea (1/18), headache (1/18), dysgeusia (1/18) and chills (1/18), were observed in five patients (20.8%) [176].

Unfortunately, after receiving QIDP and Fast Track designation for ABSSSIs and CABP in the United States in 2013, there have been no new studies or updates for avarofloxacin. This is possibly related to the acquisition of Furiex Pharmaceuticals in July 2014 by Actavis, now known as Allergan plc. [177].

### 4.3. Polymyxin Derivatives

#### SPR-741

SPR-741, developed by Spero Therapeutics, is a novel, polycationic, polymyxin derivative that is capable of potentiating the activity of various antibiotics [178]. Polymyxins are bactericidal antibiotics that permeabilise the outer membrane, whose action is restricted to Gram-negative bacteria [179]. However, when SPR-741 was used in combination with retapamulin, a semisynthetic, pleuromutilin antibiotic typically active against Gram-positive bacteria, the MIC of retapamulin was reduced 256-fold to 0.03 μg/mL [180]. SPR741 was specifically designed to minimise nephrotoxicity, and thus has a reduced positive charge and does not have the highly lipophilic fatty-acid side chain usually present in polymyxins [181].

A phase I study was conducted in 2017 to assess the safety, tolerability and pharmacokinetics of single and multiple intravenous doses of SPR-741 in healthy adult volunteers (NCT03022175) [182]. SPR-741 demonstrated a peak of mean plasma concentrations one hour after a single intravenous dose which declined over 24 h [183]. A linear and proportional pharmacokinetic profile was associated when a single one hour intravenous infusion at doses up to 800 mg was administered [183]. SPR-741 was found to have a half-life ranging from 2.0 to 3.8 h with no evidence of accumulation or time-dependent changes in plasma exposure [183]. In the presence of other antibiotics, there was no increase or decrease in the clearance or half-life of SPR741, suggesting a lack of drug–drug interaction [183]. Both single and multiple doses of SPR741 was well tolerated in healthy adult subjects [183]. In the single-ascending dose part, 34 treatment-emergent adverse events were reported in 15/48 (31%) of the subjects, compared to 5/16 (31%) in placebo-treated subjects [183]. The most common treatment-emergent adverse event was headache (8%) in four subjects, all of which were unrelated to the study drug [183]. There was no evidence of abnormal serum creatinine values and no subject experienced a renal or urinary disorder [183]. In the multiple-ascending dose section, all subjects experienced at least one adverse event with the most common being headache (33%), contact dermatitis (29%), decreased creatinine clearance (25%) and diarrhoea (13%) [183]. Renal clearance decreased on Day 14 with an increasing dose, suggesting renal elimination in saturated at higher repeat doses [183].

The results of this proof-of-concept study warrant further preclinical investigation of antibiotic combinations to determine its efficacy against bacterial species [181].

### 4.4. Clostridium Difficile Infections (CDI)

#### 4.4.1. Ridinilazole

Ridinilazole, previously known as SMT19969, is a novel antibacterial agent developed by Summit Therapeutics, for the treatment of *Clostridium difficile* infections (CDI) [184]. Ridinilazole demonstrates specific activity against *C. difficile* and has demonstrated lower propensity of affecting the gut microbiome due to limited activity against gut microbiota and diminished production of *C. difficile* toxins [184]. It has a unique mechanism of action, interfering with cell division.

In vitro, the pharmacodynamic effects of ridinilazole have been tested against *C. difficile* strains, demonstrating an MIC90 of 0.125 mg/L, 16 to 32 times lower than metronidazole and vancomycin [184]. The in vitro production of toxins A and B in C. *difficile* strains was also examined, resulting in the suppression of toxin B production below the limit of detection, and a reduction in toxin A levels by 75–90% at various ridinilazole concentrations [184]. Comparatively, metronidazole and vancomycin have limited impact on toxin production, conferring its potential as an effective treatment for CDI [184]. In hamster models of CDI, ridinilazole has been shown to be superior to vancomycin, protecting from initial infection and recurrent disease [185].

A phase I study investigating the safety and pharmacokinetics following single and multiple oral doses up to 2000 mg of Ridinilazole was conducted in 56 healthy male subjects [185]. All doses were well tolerated as single oral doses or twice daily oral doses for 10 days [185]. Possibly due to its highly selective nature, there was minimal disruption to the normal microbiota [185]. Repeat dosing suggests increased absorption over time, as the proportion of subjects with measurable plasma levels on Day 10 was greater. Phase II trials have shown that participants receiving 200 mg every 12 h of ridinilazole had a higher sustained clinical response than patients receiving 125 mg every six hours of vancomycin (66.7% vs 42.4%, p = 0.0004) (NCT02092935) [184]. Ridinilazole showered statistical superiority in sustained clinical response rates compared to vancomycin [186]. The most commonly reported adverse reactions were nausea (20%) and abdominal pain (12%), with the majority being gastrointestinal-related (40%), similar to that of vancomycin (56%) [184]. Another phase II, randomised clinical trial was completed in 2016, investigating the safety and efficacy of a 200 mg twice daily dose of Ridinilazole for 10 days, compared to fidaxomicin for the treatment of CDI (NCT02784002), however results have not been published [187].

Ridinilazole was designated as a QIDP and the US FDA granted its Fast Track status in 2016 [186].

#### 4.4.2. CRS3123

CRS3123 (formerly named REP3123), developed by Crestone Inc., is a novel synthetic diaryldiamine with a mechanism of action involving the inhibition of methionyl-tRNA synthetase (MetRS) in Gram-positive bacteria, with particularly excellent in vitro bacteriostatic activity against *C. difficile* [188]. Specifically, CRS3123 shows in vitro activity against a wide range of *C. difficile* strains, including the highly virulent BI/NAP1/027 strain [189]. CRS3123 has a narrow spectrum of antibacterial activity, notably being inactive against numerous species of Gram-positive intestinal colonisers such as Lactobacillus and Bifidobacterium, and thus poses less risk of disrupting the intestinal ecological balance [188,189]. Moreover, an in vivo hamster model study demonstrated its superior inhibitory activity of de novo synthesis of *C. difficile* toxins and sporulation compared to vancomycin and metronidazole [190]. CRS3123 exhibits no activity against normal flora gut anaerobes and Gram-negative bacteria, theoretically maintaining the colonisation resistance barrier [188].

The first human study of CRS3123 is a double-blind, randomised, placebo-controlled phase I trial evaluating the safety and pharmacokinetics of CRS3123 single oral dose therapy of either 100, 200, 400, 800 or 1200 mg in healthy adults (NCT01551004), which found similar severity and frequency of adverse effects for CRS3123 and placebo treatment groups [191]. The commonest adverse events in the CRS3123 group were decreased haemoglobin (23.3%), headache (20%), abnormal urine analysis (20%), and positive urine leukocyte esterase (16.7%), all of which were rated as mild to moderate [191]. No allergic or anaphylactic reactions were found [191]. Pharmacokinetic data show that its plasma concentration peaks at two to three hours after dosing and rapidly declines over 12 h, and that systemic absorption occurs only at higher doses which are supratherapeutic [191]. As the formation of glucuronides is likely in CRS3123, this has the potential to increase renal excretion [191]. However, absorbed CRS3123 was excreted in the urine in both its native state and after glucuronidation, so modification does not seem to be required for renal clearance [191]. Glucuronides are unlikely to result in increased toxicity or biological activity as they are rarely biologically active [191]. Furthermore, due to a lack of analytical standards for unexpected metabolites, the study was unable to accurately assess the oral bioavailability of CRS3123 in humans [191]. This molecule is currently undergoing phase II trial development for *C. difficile* infections in the United States [192].

#### 4.4.3. DS-2969

DS-2969, developed by Daichi Sankyo Co., Ltd., is a novel DNA gyrase B (GyrB) inhibitor intended for treating C. difficile infection (CDI) [193]. There are two forms of DS-2969: DS-2969a is the free form of DS-2969b that is actively secreted into the colon for faecal excretion [193], while the specific mechanism of action for DS-2969b involves binding to ATP-binding site of DNA gyrase, which is distinctive from the quinolone-binding site, hence DS-2969b showed no cross-resistance with other antibacterial agents in in vitro studies [193].

In a neutropenic murine MRSA lung infection model, DS-2969b (50 and 100 mg/kg/day) demonstrated bactericidal potential at lower doses than linezolid (four-fold higher dose)and vancomycin (220 mg/kg/day) [194]. DS-2969b exhibited time-dependent slow killing of MRSA with a MIC90 of 0.25 μg/mL, which is eight-fold lower than linezolid [194]. The MIC of DS-2969b was not affected by the presence of a pulmonary surfactant [194].

In a hamster CDI model, DS-2969b demonstrated the most potent in vitro activity against C. difficile isolates compared to fidaxomicin, vancomycin, and metronidazole (MIC90 of 0.06, 0.125, 2, and 1 μg/mL, respectively), while preserving a greater amount of intestinal microbiota bifidobacterium compared to vancomycin [193]. Furthermore, DS-2969 demonstrated superior activity against the NAP1/027 strain of C. difficile compared to vancomycin or fidaxomicin [193]. One pharmacological property of potential concern for DS-2969b is its much higher relative oral bioavailability and systemic exposure compared to vancomycin and fidaxomicin, with up to an estimated 70% in an in vivo study featuring cynomolgus monkeys [193,195].

A more recent 2018 phase I study, evaluating the safety, tolerability, pharmacokinetic and pharmacodynamic properties of daily oral ascending doses of DS-2969b in healthy human adults, demonstrated its safety in all dose cohorts, including 400 mg of oral daily doses for up to 14 days [195]. Plasma half-life of DS-2969b was determined to be approximately 15 h, with mainly renal excretion, but also via faecal routes [195]. Drug-related adverse reactions occurred in three subjects given the 400 mg dose [196], with the commonest treatment-emergent adverse events being constipation (1/18, 5.6%), abdominal bloating (1/18, 5.6%), left lower quadrant abdominal pain with hematochezia (1/18, 5.6%), and diarrhoea (1/18, 5.6%), with no dose-effect relationship identified [195,196].

To date, there are no registered clinical trials for DC-2969 in the United States.

#### 4.4.4. MGB-BP-3

MGB-BP-3, currently under development by MGB Biopharma, is the first agent in a new chemical class known as DNA minor groove binder [197]. It is a structural derivative of distamycin [198]. Specifically, its mechanism of action involves the interference of C. difficile DNA transcription by selectively binding to the AT-rich regions along the DNA minor groove, which is made possible by the concave-shaped aromatic framework of its chemical structure [199]. MGB-BP-3 has bactericidal activity against Gram-positive species including MSSA, MRSA, streptococci including S. pneumoniae, vancomycin-resistant and -susceptible enterococci and C. difficile [200].

Since September 2016, MGB-BP-3 has been granted QIDP designation for the treatment of C. difficile-associated diarrhoea (CDAD) [201]. To date, there is one phase I clinical trial evaluating the safety, blood levels and effects of MGB-BP-3 in healthy human adults (NCT02518607) [202]. This single-centre, double-blind, placebo-controlled study found that single and repeated doses of up to 2000 mg per day were well tolerated, and the drug had undergone no systemic absorption as it was undetectable in any plasma or urine samples [200]. The investigators reported no serious adverse events were noted in the single- or multiple-dose escalation study [200]. Treatment-emergent adverse events were reported by 16.7–33.3% of subjects at all dose levels of MGB except at doses of 250 mg and 2000 mg [200]. Plasma and urine pharmacokinetic samples at all doses did not detect MGB, however, all faecal samples collected at 48–72 h after 1000, 1500 and 2000 mg and in 33%, 40% and 67% after doses of 250, 500 and 750 mg, respectively, were detected [200]. MGB was found to remain in the gastrointestinal system after oral administration with no systemic absorption [200].

An exploratory phase IIa open-label study that assessed the safety, tolerability, and efficacy of MGB-BP-3 in adult patients with CDAD has obtained its NCT number (NCT03824795) and is currently recruiting [203]. As of January 2019, the FDA has granted the Investigational New Drug status to MGB-BP-3 for its use in CDAD [204].

### 4.5. Other Antibiotic Categories

#### 4.5.1. KBP-7072

KBP-7072, developed by KBP Biosciences, is a novel aminomethylcycline antibiotic aimed at combatting CAP [205]. Although information on its exact mechanism of action and spectrum of antibacterial activity is limited, it is known as a third-generation tetracycline with a broad spectrum that covers Gram-positive and Gram-negative bacterial pathogens [206].

Two ascending-dose pharmacokinetic studies involving both single and multiple doses, have demonstrated a relatively prolonged half-life (25 to 46 h) [206,207]. In a neutropenic murine pneumonia model study, KBP-7072 showed in vivo efficacy against S. aureus and S. pneumoniae strains that exhibit higher minocycline MIC and beta-lactam resistance [205]. The pharmacokinetic measurements were linear over a dose range of 1 mg/kg to 256 mg/kg of body weight subcutaneously [205]. The penetration into the epithelial lining fluid ranged from 82% to 238% compared to plasma free drug concentrations [205]. Elimination half-life was found to range from 3.2 to 4.6 h with relatively linear maximum concentration of drug in serum over the dose range [205].

To date, two phase I clinical trials were completed evaluating the safety, tolerability and pharmacokinetic properties of KBP-7072 in healthy adults (NCT02454361 and NCT02654626) [208,209]. In the multiple ascending dose study, KBP-7072 was found to be tolerated at up to 200 mg in a once daily oral administration for ten days with the therapeutic dose likely to be less than 200 mg/day. The commonest treatment-emergent adverse events were elevated ALT (4/16) [206]. However, the elevations are mild, asymptomatic and the patients recovered without any interventions [206]. KBP-7072 is currently being developed for oral administration [210].

As of November 2016, FDA has granted KBP-7072 both QIDP and Fast Track designations for the treatment of CABP [211].

#### 4.5.2. Teixobactin

Teixobactin, developed by Novobiotics Pharmaceuticals, is a macrocyclic depsipeptide hypothesised to be synthesised by Eleftheria terrae [212]. Its mechanism of action is primarily by binding to lipid II and III, precursors of peptidoglycan and cell wall teichoic acid, respectively [212]. This inhibits the production of the peptidoglycan layer and leads to the lysis of vulnerable bacteria [212].

In vitro, teixobactin had excellent activity against Gram-positive pathogens, including drug-resistant strains, with potency against most species [212]. Teixobactin was superior to vancomycin in killing late exponential phase populations of *S. aureus* as well as retained bactericidal activity against intermediate resistance *S. aureus* [212]. Resistant mutants of *S. aureus* were not producible even after serial passage of *S. aureus* when administering sub-lethal doses over 27 days [212]. Furthermore, there was no toxicity against mammalian NIH/3T3 and HepG2 cells at the highest dose tested and no haemolytic activity or DNA binding was observed, suggesting that teixobactin is a peptidoglycan synthesis inhibitor [212].

Another in vitro study that evaluated three synthesised derivatives of teixobactin to determine their activity against both Gram-positive and Gram-negative bacteria also demonstrated potent antimicrobial activity against Gram-positive bacteria [213]. Both sensitive Gram-positive MRSA and VRE isolates were inhibited, to a superior level than that of vancomycin [213]. The strong bactericidal activity may be accredited to the synergistic inhibition of the synthesis of both peptidoglycan and cell wall teichoic acid [213].

In mouse septicaemia models infected intraperitoneally with MRSA, teixobactin had a protective dose for 50% of the population (PD50) of 0.2 mg/kg compared to that of vancomycin, the chief antibiotic used to treat MRSA (2.75 mg/kg) [212]. Similar results were obtained in a thigh model of infection with *S. aureus* [212]. In mice infected with *S. pneumoniae*, teixobactin also showed good efficacy, resulting in a 6log10 reduction in colony-forming units in the lungs [212].

In 2018, a synthetic version of teixobactin was developed and trialled both in vitro and in vivo [214]. In mouse models of infectious keratitis, the severity of corneal oedemas was reduced significantly compared to moxifloxacin-treated corneas [214]. No clinical trials are in current development as of 2019.

#### 4.5.3. TP-271

TP-271, developed by Tetraphase Pharmaceuticals, is a novel synthetic derivative of tetracyclines designed for the treatment of complicated bacterial respiratory infections caused by both Gram-positive and Gram-negative pathogens [215]. TP-271 acts on the 30S ribosomal subunit, preventing the binding of aminoacylated tRNA to the A site, thus inhibiting new amino acid addition and peptide chain growth [216].

In vivo, TP-271 was active against Gram-positive pathogens including *S. pneumoniae* (MIC90 = 0.03 µg/mL), MSSA (MIC90 = 0.25 µg/mL), MRSA (MIC90 = 0.12 µg/mL) and *S. pyogenes* (MIC90 = 0.03 µg/mL) [215]. Compared to tetracycline, TP-271 was ≥1000-fold more potent against *S. pneumoniae* and *S. pyogenes*, and 128-fold more potent than *S. aureus* [215]. TP-271 also displayed good antibacterial potency against drug-resistant pathogens, including *S. pneumoniae* resistant to penicillin and macrolides, and MRSA displaying resistance to fluoroquinolones, macrolides, linezolid and daptomycin [215]. Both intravenous and oral administration of TP-271 in rodent pneumonia models demonstrated efficacy against MRSA, *S. pneumoniae* and *H. influenzae*, making it a promising antibacterial drug for the treatment of CABP [215].

Two phase I studies have been completed with pending results, with one assessing the safety, tolerability and pharmacokinetics of intravenous TP-271 in healthy adults (NCT02724085), and another studying the multiple ascending dose of oral TP-271 in healthy adult subjects (NCT03450187) [217,218]. Two other phase I clinical trials assessing the pharmacokinetics of oral (NCT03024034) and intravenous (NCT03234738) TP-271 are currently active, but are not recruiting [219,220].

## 5. Promising Alternative Treatment Approaches

### 5.1. Bacteriophages

#### AB-SA01 and Other Bacteriophages

The rise of antibiotic resistance has led to renewed interest in bacteriophage (phage) therapy, which has been around for almost a century [221]. Emerging biotechnological advances, such as bioengineered phages and purified phage lytic proteins, have the potential to act as an alternative to antibiotics due to their ability to invade and lyse bacteria at the infection site [221]. Phages are highly specialized and targeted for bacteria, and therefore are unable to infect mammalian cells [222]. This results in decreased toxicity and adverse events, making it a promising alternative to antibiotics.

AB-SA01, developed by Armata Pharmaceuticals, is a highly characterised phage cocktail of three naturally occurring lytic *S. aureus* phages, designed and developed to treat *S. aureus* infections, especially those caused by MRSA [222]. The three component phages have not been shown to contain identifiable genes related to bacterial virulence or antibiotic resistance [222]. In vitro, AB-SA01 demonstrated activity to 94.5% of 401 clinical *S. aureus* isolates, including sensitivity to 95% of the total 205 MDR isolates [222]. No evidence of interference among the component phages was observed with the *S. aureus* strains. AB-SA01 also demonstrated activity against two of five S. epidermis strains but no cross-genus activity [222]. In vivo, AB-SA01 showed efficacy similar to that of vancomycin in two murine lung infection models [222]. In both models, *S. aureus* colonies showed sensitivity to AB-SA01 and there was no evidence of phage-resistant colonies [222].

A phase I trial evaluating the safety, tolerability and preliminary effectiveness of AB-SA01, in nine patients with chronic rhinosinusitis associated with *S. aureus* infection (ACTRN12616000002482) [223]. The patients were separated into three cohorts, each receiving different doses of AB-SA01 of varying duration (3 × 108 plaque-forming units (PFU) for 7 days, 3 × 108 PFU for 14 days, and 3 × 109 PFU for 14 days) [223]. Intranasal phage treatment was well tolerated, with six reports of mild treatment-emergent and no serious adverse events reported [223]. Six adverse effects were reported in six participants, including loose bowels (1/3), self-resolved epistaxis (2/3), symptoms of upper respiratory tract infection (2/3), oropharyngeal pain (2/3), rhinalgia (2/3) and low serum bicarbonate level (2/3), with rhinalgia being the only adverse event likely to be related to the phage therapy [223]. Laboratory tests for liver, kidney and haematology function were within the normal limits [223]. In all three cohorts, preliminary efficacy results indicated favourable outcomes, with two patients showing clinical and microbiological evidence of infection eradication [223]. Normal *E. coli* flora was not found to have decreased and oral phage treatment did not have an effect on the faecal microbiota composition [223]. Furthermore, as phages self-replicate at the site of infection, this reduces the need for frequent administration [223]. At three-month follow up, four patients, including two with *S. aureus* eradication, showed a continuing improvement in all outcome measures compared to pre-treatment [223]. The study concluded that twice-daily intranasal irrigations to 3 × 109 PFU for 14 days was safe, well-tolerated and had no dose-limiting adverse effects [223].

In September 2018, it was announced that the FDA agreed with the company’s trial designs for phase I/II clinical trials, set to begin in 2019 [224]. The proposed trial will investigate the safety, tolerability and efficacy of intravenous administration of AB-SA01 as an adjunct to existing antibiotic therapy for the treatment of ventricular assist devices infected by *S. aureus* [225]. In addition, the role of AB-SA01 as an adjunct to surgical treatment in patients with a hip or knee prosthetic joint infection due to *S. aureus* will also be investigated [224]. An open-label, single-arm study has already evaluated the administration of AB-SA01 intravenously, as adjunctive treatment to antibiotics, in 13 critically ill patients with *S. aureus* bacteraemia (including six with endocarditis), in one centre in Australia [226]. No safety concerns were raised, and efficacy analyses showed a marked reduction in staphylococcal DNA in blood as well as decrease in inflammatory markers [226].

A randomised, placebo-controlled phase II and III clinical trial investigating the use of other bacteriophages in the treatment of UTIs (NCT03140085) involving uropathogens, such as Staphylococcus and Streptococcus species, was completed in 2018, with the results still pending [227]. Currently, a randomised, multi-centre, controlled phase I/II interventional trial is underway to compare the efficacy of standard treatment associated with phage therapy versus placebo for diabetic foot ulcers infected with MRSA and MSSA(NCT02664740) [228]. The study is expected to be completed in August 2019; however, it has not yet started recruiting [228].

There is a Polish interventional study which began in 2005, which aimed to investigate experimental phage therapy for non-healing post-operative wounds or drug-resistant bacterial bone, upper respiratory tract, genital or urinary tract infections (NCT00945087) [229]. Its recruitment status is currently unknown with the last update in 2013 [229].

### 5.2. Monoclonal Antibodies

#### 5.2.1. CAL02

CAL02, developed by Combioxin SA, is a novel antitoxin liposomal agent which consists of a mixture of liposomes, and acts as a toxin trap for a large range of bacterial toxins [230]. Due to the neutralising role of CAL02, it has the potential to protect against toxin-mediated organ damage and inflammation [230].

In vitro data suggest that the major decrease in bacterial load in infected animals after CAL02 was injected was due to the defence of host immune cells from lysis by bacterial toxins [231]. In vivo, treatment by CAL02 significantly decreased bacterial loads in infected mice and there was no evidence of modified bacterial killing by mouse blood or activation of neutrophils to release nitric oxide [231]. The study found substantially improved survival outcomes in mice with severe pneumonia and bacteraemia when CAL02 is combined with antibiotics [231]. Neither low doses of 12.5 mg/kg/injection of CAL02 nor 100 mg/kg/injection of vancomycin were sufficient to protect the mice from a systemic *S. aureus* infection; however, the combination of both was able to provide complete protection [231]. This combination was also most effective against fatal sepsis caused by *S. pneumoniae* [231]. Pneumococcal loads in blood 24 h after infection were substantially reduced compared to antibiotics alone and signs of disease in mice with *S. pneumoniae* infections were completely eradicated [231].

The first-in-human, dose-escalation, randomised trial was conducted in patients with severe CAP infected with *S. pneumoniae* to assess the safety and tolerability of intravenous low-dose (4 mg/kg) and high-dose (16 mg/kg) CAL02 (NCT02583373) [230]. More favourable patient outcomes were observed when treated with high-dose CAL02 compared with placebo, as 56% vs. 20% in the treatment and placebo arm, respectively, were cured on day eight [230]. Both low- and high-dose CAL02 had faster improvements of organ dysfunction, with a 50% improvement in SOFA score by day five, compared with 12.5% in the placebo group [230]. Adverse events occurred in 86% (12/14) of patients in the CAL02 treatment groups combined and 100% (5/5) in the placebo group, with 124 treatment-emergent adverse events in total [230]. The treatment-emergent adverse events in the CAL02 group resolved in 67% (57/85) of cases in the CAL02 group and 82% (32/39) resolved in the placebo group [230]. No serious adverse events were reported in relation to the study drug [230]. Adverse events that occurred in the CAL02 group, but not in the placebo group, include anaemia (21%), thrombocytopaenia (21%), pleural effusion (36%) and hypoglycaemia (21%); however, the most frequently reported adverse events were linked to underlying disease [230]. The entrapped toxins that remain following the degradation of CAL02 in the liver is a crucial factor in the safety of CAL02; however, no differences in hepatocellular injury were observed between the CAL02 group and the placebo group [230]. Overall, no parameter was worse with CAL02 and both low- and high-dose CAL02 were found to be safe and well-tolerated [230]. CAL02 does not have any of the risk factors associated with some liposomal formulations, such as hypersensitivity-related changes in blood pressure and ECG at the first exposure [230]. Due to the safety profile, tolerability and activity against a broad range of secreted toxins, CAL02 has potential benefit as an adjunctive empirical therapy.

#### 5.2.2. AR-301 (Formerly KBSA301)

AR-301 (trade name Salvecin), developed by Aridis Pharmaceuticals, is a fully human monoclonal antibody that specifically neutralises alpha toxins, produced by *S. aureus* [232,233]. Alpha-toxin is a key virulence factor of *S. aureus* and can lead to tissue disruption, bacterial dissemination, immune dysregulation and programmed cell death when released into the infected host cell [234,235]. Alpha toxin has also recently been shown to modulate the activity of macrophages in co-infecting pathogens, including *P. aeruginosa* and *Klebsiella pneumoniae* [232]. Therefore, the neutralisation of alpha toxins should prevent damage caused by the toxin and bacteraemia [236]. Unpublished data have shown AR-301 to be protective both prophylactically and therapeutically against *S. aureus* [232].

A first-in-human, single-ascending dose, phase I/IIa study was conducted to investigate the safety and tolerability of AR-301 as an adjunctive therapeutic treatment to standard antibiotics in 48 patients with severe *S. aureus* pneumonia (NCT01589185) [232]. AR-301 was administered via intravenous infusion over two hours, starting within 36 h of the diagnosis of severe pneumonia. The pharmacokinetic profile of AR-301 is consistent to that of a human IgG1 monoclonal antibody, with a half-life of approximately 25 days [232]. AR-301 (22 patients (71%)) was not statistically different compared to placebo (14 patients (87.5%)) in terms of rate of clinical cure on Day 28 [232]. Similarly, the duration of ventilation was slightly shorter for VABP, HABP and CABP patients treated with AR-301 than placebo; however, it was not significantly different [232]. The total duration of hospital stay and duration of ICU stay was also not statistically different between the AR-301 (21.2 and 14.8 days, respectively) and placebo group (23.9 and 16.5 days, respectively) [232]. Microbiological eradication was similar in both groups and the time for *S. aureus* eradication was shorter in AR-301 treatment groups; however, the difference was not statistically significant [232]. At least one adverse event was reported in 46 patients, with a total of 343 events, eight (or 2.3%) of which were deemed treatment-related by the investigator [232]. Treatment-related adverse events included increased LDH (2.1%, 1/48), increased eosinophil count (2.1%, 1/48) and hepatic enzymes (2.1%, 1/48), vomiting (2.1%, 1/48), fever (2.1%, 1/48), hepatocellular injury (2.1%, 1/48), arthritis (2.1%, 1/48) and plasma cell myeloma (2.1%, 1/48), which occurred in a total of six patients [232]. Most adverse events were mild or moderate and (36 or 10.5%) serious; however, none of the serious adverse events were considered treatment-related [232]. Overall, AR-301 resulted in better and faster eradication by day 28 [232].

A multi-centre, prospective, phase III trial is currently recruiting patients to evaluate the use of AR-301 as an adjunctive treatment of VAP due to *S. aureus* with standard of care antibiotic therapy (NCT03816956) [237]. This study has an estimated completion date of August 2020 [237].

#### 5.2.3. ASN-100

ASN-100, previously developed by Arsanis Inc. (now X4 Pharmaceuticals) is an investigational monoclonal antibody, involving a combination of two co-administered human monoclonal antibodies, ASN-1 and ASN-2 [238]. ASN-100 neutralises six cytotoxins released by *S. aureus*: α-haemolysin, H1gAB, H1gCB, LukED, LukSF and LukGH leucocidins, which inhibit the cytolytic activity of *S. aureus* towards human cells in vitro [238].

The safety, tolerability and serum and lung pharmacokinetics of ASN-100 was investigated in a single-dose escalation, first-in-human study with 52 healthy volunteers [239]. ASN100 was administered intravenously as a 1:1 ratio of ASN-1 and ASN-2 simultaneously through separate intravenous lines at 3600 or 8000 mg [239]. ASN100 demonstrated linear serum pharmacokinetics with a half-life of approximately three weeks and measurable levels of ASN-1 and ASN-2 were detected in ELF from as early as day one to day 30 post-dosing [239]. No dose-limiting toxicities or treatment-emergent anti-drug antibody responses were detected [239]. All adverse events resolved without treatment and the frequency of these events was not dependent on dosages [239].

Another phase II, randomised placebo-controlled study was conducted in approximately 65 sites to determine the safety, tolerability and efficacy of a single dose of ASN-100 for the prevention of pneumonia in heavily colonized, mechanically ventilated patients (NCT02940626) [240]. However, this study was terminated as a result of a pre-planned interim analysis for futility [240]. The incidence of *S. aureus* pneumonia was well below the expected rate based on previous reports and the effect by ASN-100 could not be adequately demonstrated in this study [241]. ASN-100 was safe and well-tolerated in an ICU population; however, the reduction in all bacterial pneumonias was found to be not statistically significant [241].

## 6. Conclusions

Several new agents shape the future of Gram-positive agents. Clinical syndromes like ABSSI and CAP, as well as pathogens like Streptococci (including enterococci) and Staphylococci (most importantly Methicillin resistant strains), are targeted by the new antibiotics. Important treatment challenges remain including the expansion of resistance to important pathogens like *C. difficile*, as well as the changing epidemiology of multi-drug resistant organisms (MDROs) (e.g., *C. diff*) and community acquisition. Many recent studies have focused on the interplay of epidemiology and risk factors associated with a disease, and the presence of an MDRO that, together with the rapid institution of a specific biomarker/diagnostic of infection, will lead to the early administration of an appropriate antimicrobial. The addition of novel agents with anti-Gram-positive activity and a favourable safety profile will be based on carefully performed prospective randomized trials that will evaluate new agents face to face with available choices. Novel approaches such as antibody use and phage therapy should be examined in the same context alone or in combination with traditional strategies. These studies will delineate the use of such novel antibiotics in the near and the long-term future.

## Figures and Tables

**Table 1:** Summary of antibiotics with activity against Gram-positive bacteria with New Drug Application (NDA) filed or are currently in Phase III clinical trials.

Drug Name	Phase	Company	Drug Class	Spectrum Against Gram-Positive Bacteria	Potential Indication	Ongoing Clinical Trials (ClinicalTrial.gov No.)
**Iclaprim**	NDA filed	Roche	dihydrofolate reductase inhibitor	MRSA, vancomycin-intermediate and vancomycin-resistant, and macrolide-, quinolone- and trimethoprim-resistant strains	ABSSSI	
**Cethromycin**	NDA filed	Abbott Laboratories (acquired by Advanced Life Sciences Inc.)	ketolide	telithromycin-resistant *S. pneumoniae*	CABP	
**Solithromycin**	Phase III	Cempra Pharmaceuticals	fluoroketolide	MRSA and macrolide-resistant *M. pneumoniae*	CABP	
**Contezolid (MRX-1)**	Phase III	MicuRx Pharmaceuticals, Inc.	oxazolidinone	MRSA, penicillin-resistant and penicillin-intermediate *S. pneumoniae*, and VRE	ABSSSI	
**Contezolid Acefisamil (MRX-4)**	Phase III	MicuRx Pharmaceuticals, Inc.	oxazolidinone	MRSA, VRE	MRSA & VRE infections in hospital setting	NCT03747497
**Lascufloxacin**	NDA filed	Kyorin Pharmaceutical Co., Ltd.	fluoroquinolone	MRSA, *S. epidermidis*, *E. faecalis*, *S. pyogenes*, *S. agalactiae*, and penicillin-resistant *S. pneumoniae*	CABP; URTI	
**Nemonoxacin (Taigexyn) ^1^**	Phase III	TaiGen Biotechnology Co., Ltd.	non-fluorinated quinolone	MRSA, multidrug-resistant *S. pneumoniae* and vancomycin-resistant pathogens	CABP; ABSSSI	NCT02840812
**Levonadifloxacin (WCK771)**	Phase III	Wockhardt Ltd.	fluoroquinolone	MRSA and staphylococci resistant to levofloxacin and moxifloxacin	ABSSSI; HAP	
**Zabofloxacin (DW-224a) ^2^**	Phase III	Dong Wha Pharmaceutical Industry Ltd.	fluoroquinolone	MRSA, methicillin-resistant coagulase-negative staphylococci, *S. pyogenes*, *E. faecalis* and *S. pneumoniae*	CABP	
**Brilacidin (PMX30063)**	Phase III	Innovation Pharmaceuticals Inc.	defensin mimetic	*S. aureus* and *S. epidermidis*	ABSSSI	

^1^ Nemonoxacin has been approved for treating community-acquired pneumonia in adults in Taiwan and China. ^2^ Zabofloxacin has been approved for treating acute exacerbations of chronic obstructive pulmonary disease in South Korea. Abbreviations: ABSSSI (Acute Bacterial Skin and Skin Structure Infection), CABP (community-acquired bacterial pneumonia), HAP (hospital-acquired pneumonia), NDA (New Drug Application), URTI (upper respiratory tract infection). Bacteria abbreviations: *E. faecalis* (*Enterococcus faecalis*), *M. pneumoniae* (*Mycoplasma pneumoniae*), MRSA (methicillin-resistant *Staphylococcus aureus*), *S. agalactiae* (*Streptococcus agalactiae*), *S. aureus* (*Staphylococcus aureus*), *S. epidermidis* (*Staphylococcus epidermidis*) *S. pneumoniae* (*Streptococcus pneumoniae*), *S. pyogenes* (*Streptococcus pyogenes*), VRE (vancomycin-resistant enterococcus).

**Table 2:** Summary of antibiotics with activity against Gram-positive bacteria currently in Phase II and Phase I clinical trials.

Drug Name	Phase	Company	Drug Class	Spectrum Against Gram-Positive Bacteria	Potential Indication	Ongoing Clinical Trials (ClinicalTrial.gov No.)
**Razupenem**	Phase II	Protez Pharmaceuticals	carbapenem	MRSA, penicillin-resistant *S. pneumoniae*, VRE and ampicillin-resistant *H. influenzae*	cSSSI	
**Tomopenem (CS-023)**	Phase II	Daiichi Sankyo Research Laboratories	carbapenem	MRSA and methicillin-susceptible *S. epidermidis*	Gram-positive bacterial infections	
**Radezolid (RX-1741)**	Phase II	Melinta Therapeutics, Inc.	oxazolidinone	*S. aureus*, *S. pneumoniae* and enterococci	CABP and bacterial vaginosis	
**Gepotidacin**	Phase II	GlaxoSmithKline	novel bacterial topoisomerase II inhibitor	MRSA, levofloxacin-resistant and multidrug-resistant *S. aureus*	ABSSSI	NCT04010539, NCT04079790, NCT04020341
**Debio1450 (AFN-1720)**	Phase II	Debiopharm	Fabl inhibitor	MRSA	ABSSSI	NCT03723551
**CG400549**	Phase II	CrystalGenomics Inc.	Fabl inhibitor	MRSA	infections caused by MRSA and VRSA	
**Ridinilazole (SMT19969)**	Phase II	Summit Therapeutics	new class-interferes with cell division	*C. difficile*	*C. difficile*-associated infections	NCT03595553, NCT03595566
**ME1100 (Habekacin) ^1^**	Phase I	Meiji Seika Pharma Co. Ltd.	aminoglycoside	MRSA, gentamicin-resistant and vancomycin-resistant *S. aureus*	sepsis and pneumonia caused by MRSA	
**Alalevonadifloxacin (WCK2349)**	Phase I	Wockhardt Ltd.	fluoroquinolone	MRSA, *S. pneumoniae*, *H. influenzae*, and *M. catarrhalis*	ABSSSI, CABP and HAP caused by MRSA	
**Avarofloxacin (JNJ-Q2 or acorafloxacin)**	Phase I	Furiex Pharmaceuticals (now Allergan plc.)	fluoroquinolone	MRSA, fluoroquinolone-resistant *S. pneumoniae*	ABSSSI; CABP	
**SPR-741**	Phase I	Spero Therapeutics	polymyxin	Not specified	Gram-positive bacterial infections	
**CRS3123 (REP3123)**	Phase I	Crestone Inc.	diaryldiamine	*C. difficile*	*C. difficile*-associated infections	
**DS-2969**	Phase I	Daichi Sankyo Co. Ltd.	DNA gyrase B inhibitor	*C. difficile*	*C. difficile*-associated infections	
**KBP-7072**	Phase I	KBP Biosciences	tetracycline	*S. aureus* and *S. pneumoniae* strains that exhibit higher minocycline MIC and beta-lactam resistance	CABP	
**MGB-BP-3**	Phase I	MGB Biopharma	DNA minor groove binder	MRSA, *S. pneumoniae*, vancomycin-resistant enterococci and *C. difficile*	*C. difficile*-associated diarrhoea	NCT03824795
**Teixobactin**	Phase I	Novobiotics Pharmaceuticals	depsipeptide	MRSA and VRE	Gram-positive bacterial infections	
**TP-271**	Phase I	Tetraphase Pharmaceuticals	fluorocycline	MRSA, *S. pneumoniae* and *S. pyogenes*	CABP	NCT03024034, NCT03234738

^1^ ME1100 (trade name Habekacin) has been approved for treating sepsis and pneumonia caused by MRSA in Japan. Abbreviations: ABSSSI (Acute Bacterial Skin and Skin Structure Infection), CABP (community-acquired bacterial pneumonia), cSSSI (complicated skin and skin structure infection), HAP (hospital-acquired pneumonia), MIC (minimal inhibitory concentration). Bacteria abbreviations: *C. difficile* (*Clostridium difficile*), *E. faecium* (*Enterococcus faecium*), *H. influenzae* (*Haemophilus influenzae*), *M. catarrhalis* (*Moraxella catarrhalis*) MRSA (methicillin-resistant *Staphylococcus aureus*), *S. aureus* (*Staphylococcus aureus*), *S. epidermidis* (*Staphylococcus epidermidis*), *S. pneumoniae* (*Streptococcus pneumoniae*), *S. pyogenes* (*Streptococcus pyogenes*), VRE (vancomycin-resistant enterococcus).
